# *Nelumbo nucifera* leaves extracts inhibit mouse airway smooth muscle contraction

**DOI:** 10.1186/s12906-017-1674-7

**Published:** 2017-03-20

**Authors:** Xiao Yang, Lu Xue, Qingyang Zhao, Congli Cai, Qing-Hua Liu, Jinhua Shen

**Affiliations:** 1 Institute for Medical Biology and Hubei Provincial Key Laboratory for Protection and Application of Special Plants in Wuling Area of China, College of Life Sciences, South-Central University for Nationalities, Wuhan, 430074 China; 2 Wuhan Youzhiyou Biopharmaceutical Co., Ltd., 666 Gaoxin Road, Biolake, Wuhan, 430075 China

**Keywords:** Asthma, Airway smooth muscle, Tracheal relaxation, Alkaloids, Ca^2+^ channel

## Abstract

**Background:**

Alkaloids extracted from lotus leaves (AELL) can relax vascular smooth muscle. However, whether AELL has a similar relaxant role on airway smooth muscle (ASM) remains unknown. This study aimed to explore the relaxant property of AELL on ASM and the underlying mechanism.

**Methods:**

Alkaloids were extracted from dried lotus leaves using the high temperature rotary evaporation extraction method. The effects of AELL on mouse ASM tension were studied using force measuring and patch-clamp techniques.

**Results:**

It was found that AELL inhibited the high K^+^ or acetylcholine chloride (ACh)-induced precontraction of mouse tracheal rings by 64.8 ± 2.9%, or 48.8 ± 4.7%, respectively. The inhibition was statistically significant and performed in a dose-dependent manner. Furthermore, AELL-induced smooth muscle relaxation was partially mediated by blocking voltage-dependent Ca^2+^ channels (VDCC) and non-selective cation channels (NSCC).

**Conclusion:**

AELL, which plays a relaxant role in ASM, might be a new complementary treatment to treat abnormal contractions of the trachea and asthma.

**Electronic supplementary material:**

The online version of this article (doi:10.1186/s12906-017-1674-7) contains supplementary material, which is available to authorized users.

## Background

Respiratory disease is a severe health issue, it affects millions of people globally and is a serious burden for public healthcare [1]. Asthma and chronic obstructive pulmonary disease (COPD), two typical airway problems, have been linked to decreased quality of life, increased medical costs and even mortality and may be an underlying factor for lung cancer [2]. Airway smooth muscle (ASM), a particular cell type in the airway system, plays a vital role in airway obstruction during asthma and COPD. The dysfunction of ASM contributes to a series of cardinal symptoms in asthma and COPD, such as airway contractility, airway remodeling, and airway hyperresponsiveness [3–5]. Thus far, the pharmacological treatment of asthma and COPD mainly relies upon β2 agonists and muscarinic antagonists, which can affect β2-adrenergic and muscarinic receptors, respectively. Through the regulation of signal pathways related to G protein-coupled receptors, the reduction of intracellular calcium, and the subsequent opening of VDCC/NSCC, airway smooth muscle tone could be relaxed [6, 7]. However, numerous studies have indicated that currently available bronchodilators, especially β2-agonists and muscarinic antagonists, have additive effects on safety, desensitization and tolerability [8–10]. Therefore, novel drugs are urgently needed for these airway diseases.

Recently, numerous traditional Chinese medicines (TCMs) have exhibited encouraging effects on a series of chronic diseases [11–15]. Additional evidence has shown that the effective constituents of TCMs may provide a new trend to explore potential bronchodilators for asthma and COPD [16–19]. *Nelumbo nucifera*, commonly called sacred lotus, is a perennial aquatic cash crop that is widely cultivated in Asia and Africa [20, 21]. In China, the flowers, seeds, roots, leaves and rhizomes of lotus are extensively applied as ornamentation, drink, food, and as a medicinal herb [21, 22]. As a medicinal herb, different parts of this plant have exhibited diverse therapeutic benefits. In traditional therapy, lotus leaves are often listed as part of a healthy diet and are provided as a “tea drink” to improve lipid disorders, control blood pressure and lose weight. Several studies have explored the bioactive compounds extracted from lotus leaves, showing that they play more pharmacological roles in the treatment of diabetes, obesity, hyperuricemia, hepatic steatosis, lung cancer, HIV, etc. [22–29]. In our lab, a recent study showed that *Plumula Nelumbinis*, the green germ of *Nelumbo nucifera*, has been identified as providing relaxation effects in ASM [30]. Furthermore, nuciferine, a constituent of alkaloids extracted from lotus leaves, presents relaxation properties in rat mesenteric arteries [31]. Therefore, it was hypothesized that alkaloids extracted from lotus leaves can affect ASM reactivity.

To verify this hypothesis, the effects of alkaloids extracted from the leaves of *Nelumbo nucifera* were investigated in mouse ASM in the current study. The results show that high K^+^ or ACh-precontracted mouse ASM could be relaxed by AELL in a concentration-dependent manner. Further research indicates that AELL mediate relaxation through voltage-dependent Ca^2+^ channels (VDCC) and non-selective cation channels (NSCC) inhibition.

## Methods

### Reagents and Chemicals

Acetylcholine chlorides (ACh), Nifedipine, Niflumic acid (NA), and Tetraethylammonium chloride (TEA) were purchased from Sigma Chemical Co. (St. Louis, MO, USA). Other chemicals were purchased from Sinopharm Chemical Reagent Co. (Shanghai, China).

### Extraction of Alkaloids

Dry *Nelumbo nucifera* leaves were bought from Tong-Ren-Tang (Hubei, China) and identified by Associated Prof. Hong Liu (South-Central University for Nationalities, Wuhan, China) and Prof. Ding-Rong Wan (South-Central University for Nationalities, Wuhan, China). The voucher specimen of this material has been deposited in the publicly available herbarium of South-Central University for Nationalities. The deposition number is SCUN201410023. 95% ethanol were employed to enrich alkaloids in lotus leaves as previous described with some modification [32]. Briefly, lotus leaves (500 g) were air dried, pulverized and soaked in 95% ethanol for 48 h at room temperature. The samples were subsequently extracted twice by heating reflux for 2 h. The pH of pooled ethanol extracts was adjusted to 2–3 with 0.5% hydrochloric acid. After filtering, 0.1 mol/L sodium hydroxide was added into the acid solutions until the pH was 5–7. Subsequently, supernatants were collected and adjusted to pH 9–11 with 0.1 mol/L sodium hydroxide and diluted to 1 L. The solution was then dried through rotary evaporation and vacuum filtered to yield AELL. The yield of AELL was 5.8% of raw material. Before experiments, AELL was dissolved with physiological saline solution (PSS) buffer (2 mM CaCl_2_, 10 mM Glucose, 10 mM HEPES, 5 mM KCl, 1 mM MgCl_2_, 135 mM NaCl, pH 7.4).

### Animals

Six-week-old male BALB/c mice were purchased from the Hubei Provincial Center for Disease Control and Prevention (Wuhan, China). The mice were housed in a specific pathogen free (SPF) grade laboratory under a 12 h light-dark cycle with a stable temperature (20–25 °C) and humidity (50–60%). All mice animal experiments were approved and performed under the supervision of the Institutional Animal Care and Use Committee of the South-Center University for Nationalities. The license number is 2013-JS-2.

### Isometric Measurement of Tension

Mouse ASM contraction was measured in tracheal rings as previously described [30]. Briefly, tracheas were isolated from cervically dislocated mice, and connective tissues were removed. Small tracheal rings (~5 mm) were cut, and the resting tension was set to 0.5 g in a settled organ bath with PSS buffer. The experiment was initiated after the tracheal rings were equilibrated for 60 min. Then, tracheal rings were precontracted with either 80 mM K^+^ or 10^−4^ M ACh for 3 times. Following an additional 30 min rest, the isometric tension was measured under the presence of K^+^, ACh, or AELL. In the rinsing process, the trachea ring was thoroughly washed with PSS for 3 times (5 min, 5 min, 50 min, respectively) to remove AELL.

### Measurement of VDCC Currents

Mouse ASM cells were isolated as previously described [30, 33, 34]. Whole-cell Ba^2+^ currents through VDCC were measured using an EPC-10 patch-clamp amplifier (HEKA, Lambrecht, Germany). The pipette solution containing 130 mM CsCl, 10 mM EGTA, 10 mM HEPES, 4 mM Mg-ATP, 4 mM MgCl_2_, and 10 mM TEA was adjusted to pH 7.2 with CsOH. The composition of the external solution was 27.5 mM BaCl_2_, 11 mM Glucose, 10 mM HEPES, 1 mM MgCl_2_, 107 mM NaCl, and 10 mM TEA-Cl, which was adjusted to pH 7.4 with NaOH. ASM cells were patched and held at −70 mV. The currents were measured following depolarization for 500 ms from −70 to +40 mV in 10 mV increments every 30 ms.

### Measurement of NSCC Currents

The pipette solution for the measurement of NSCC currents containing 1 mM CaCl_2_, 126 mM CsCl, 3 mM EGTA, 10 mM HEPES, and 1.2 mM MgCl_2_ was adjusted to pH 7.2 with Tris. The free Ca^2+^ concentration was approximately 70 nM, as calculated using WEBMAXC STANDARD (http://www.stanford.edu/~cpatton/webmaxc/webmaxcS.htm). The bath solution was K^+^-free PSS (1.5 mM CaCl_2_, 11 mM glucose, 10 mM HEPES, 126 mM NaCl) containing 100 μM NA, 10 μM nifedipine, or 10 mM TEA, which were employed to block Cl^−^, VDCC, or K^+^ currents, respectively. ACh-induced NSCC currents were recorded with a ramp using a perforated whole-cell configuration from −80 to +60 mV over 500 ms. The holding potential was −60 mV.

### Statistical Analysis

Statistical analysis and significance were measured with Student’s t-test using Origin 9.0 software (OriginLab, Northampton, USA). The results are expressed as the means ± SEM. In all comparisons, *p* < 0.05 was considered statistically significant.

## Results

### AELL Dose-dependently Inhibit High K^+^-induced Precontraction

**Fig. 1:**
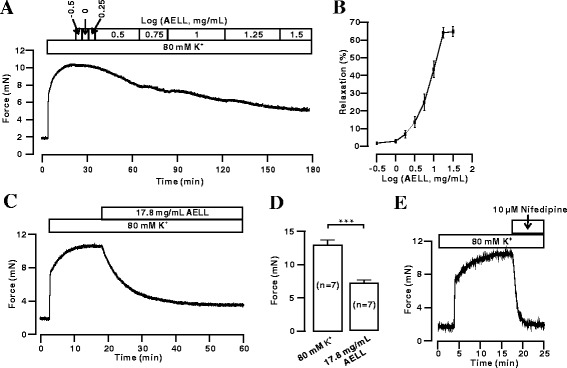
AELL inhibits high K^+^-induced precontraction of mouse ASM. **a** High K^+^ induced a contraction in a tracheal ring, which was gradually inhibited by AELL. **b** The same experiments were performed in 9 tracheal rings/9 mice, and a dose-relaxation curve was constructed. **c** & **d** High K^+^-induced precontraction was inhibited by AELL (17.8 mg/mL), which was observed in 7 tracheal rings/7 mice. **e** The same contraction was blocked by nifedipine, a selective VDCC blocker. (*n* = 5). *** represents *p* < 0.001

A high K^+^ concentration causes the contraction of smooth muscle through the membrane depolarization-VDCC open-calcium influx pathway [35, 36]. The high K^+^ (80 mM)-induced contraction was blocked by AELL in a dose-dependent manner (Fig. 1a, b). The maximal relaxation was 64.8 ± 2.9% (*n* = 8). The half-maximal inhibition (IC_50_) was 7.28 ± 1.4 mg/mL. Moreover, after AELL washing out, the high K^+^-induced secondary contraction decreased significantly (*n* = 7) (Additional file 1: Figure S1A & B). This result indicated AELL might have a long-term effect on precontracted ASM. Figure 1c and d showed that AELL (17.8 mg/mL) could relax a high K^+^-induced precontraction of ASM by 56.33% ± 6.7%. Nifedipine (10 μM), which is a selective inhibitor of VDCC, completely blocked high K^+^-induced contraction (Fig. 1e). These results indicate that AELL-induced relaxation might be due to the inhibition of VDCC.

### AELL Block High K^+^-evoked Ca^2+^ Influx

**Fig. 2:**
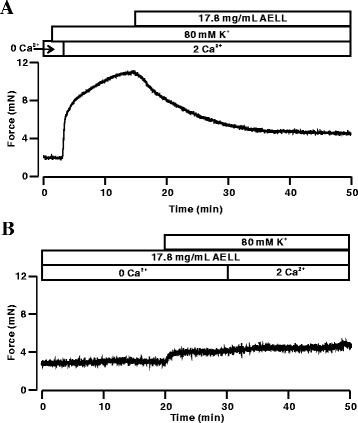
AELL blocks high K^+^-evoked Ca^2+^ influx. **a** A representative tracing of 4 tracheal rings/4 mice. Under Ca^2+^-free conditions (0 Ca^2+^ and 0.5 mM EGTA), high K^+^ did not evoke contraction in a tracheal ring. After the restoration of 2 mM Ca^2+^, a sustained contraction occurred, which was partially inhibited by 17.8 mg/mL AELL. **b** Experiments were performed as described in Fig. 2a but with the order of high K^+^ and AELL (17.8 mg/mL) addition reversed. In the presence of 17.8 mg/mL AELL, a non-significant contraction was recorded after the restoration of 2 mM Ca^2+^

The blockage of VDCC was shown to relax ASM contraction via a decrease in intracellular Ca^2+^ [37]. To confirm this, the following experiments were performed. High K^+^-induced contraction under Ca^2+^-free conditions did not occur (Fig. 2a). Following the restoration of Ca^2+^, contraction immediately appeared and was greatly inhibited by AELL (17.8 mg/mL, Figure 2a). Meanwhile, ASM contraction induced by high K^+^-evoked Ca^2+^ influx was not obvious in the presence of 17.8 mg/mL AELL (Fig. 2b). These data suggest that AELL relax mouse ASM by the partial blockage of high K^+^-evoked Ca^2+^ influx.

### AELL Block VDCC Currents

**Fig. 3:**
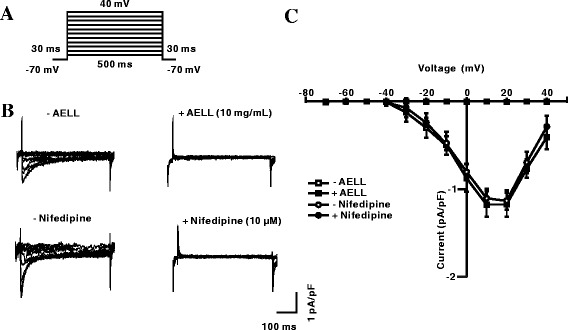
AELL blocks VDCC currents. **a** Measurement of VDCC currents following a range from −70 mV to 40 mV. **b** VDCC currents were recorded following depolarization, which were blocked by AELL or nifedipine. **c** *I-V* relationship was constructed based on the results of 5 to 6 experiments

To further clarify the contribution of VDCC currents in the ability of AELL to relax ASM, the channel-mediated currents were measured with the whole-cell patch-clamp technique. As shown in Fig. 3a, the currents were recorded from −70 mV to +40 mV. The currents were eliminated by AELL (10 mg/mL) (Fig. 3b, top). As a positive control, the currents were blocked by nifedipine, indicating that they are VDCC currents (Fig. 3b, bottom). As a type of voltage-dependent channel, current-voltage (*I-V*) curves were constructed (*n* = 6, Fig. 3c). These results suggest that AELL inhibit VDCC currents.

### AELL Inhibit ACh-induced Precontraction

**Fig. 4:**
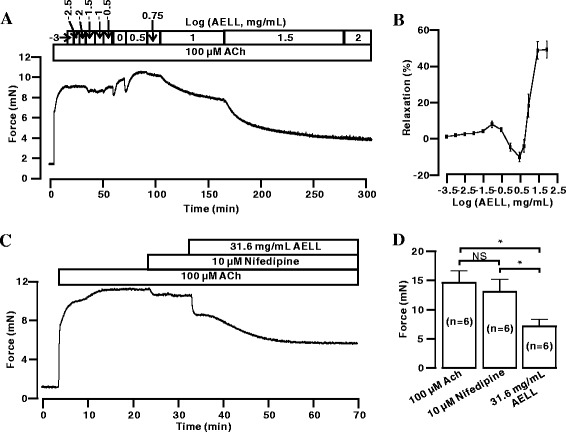
AELL inhibits ACh-induced precontraction of mouse ASM. **a** ACh induced a steady-state contraction in a mouse tracheal ring, which was inhibited by AELL in a concentration-dependent manner. **b** Dose-relaxation curve of AELL based on the results of 7 different experiments shown in (**a**). **c** ACh induced a steady contraction even in the presence of nifedipine; however, it was partially inhibited by AELL. **d** Comparison of the relaxant effects of nifedipine and AELL on ACh-induced contraction. NS represents no significance. * represents *p* < 0.05

As a particular muscarinic receptor agonist, ACh can induce the contraction of ASM through both VDCC and NSCC [38, 39]. As shown in Fig. 4a, AELL could relax ACh-induced precontraction in a dose-dependent manner. A relaxation curve was calculated, and the maximal relaxation was 48.8 ± 4.7% (Fig. 4b). As shown in Additional file 1: Figure S1C & D, the ACh-induced contraction could not recover after AELL washing out (*n* = 6). Furthermore, to identify the role of NSCC, VDCC was blocked with nifedipine. The precontraction was subtly blocked by nifedipine (10 μM). Subsequently, the isolated tension was mostly eliminated by AELL (31.6 mg/mL) (Fig. 4c). In Fig. 4d, the bar graph shows that the maximal inhibition by nifedipine and AELL was 13.32 ± 3.8% and 48.47 ± 1.8% (*n* = 5), respectively. These experiments indicate that in addition to VDCC, NSCC might also be involved in AELL-induced relaxation.

### AELL Block ACh-evoked Ca^2+^ Influx

**Fig. 5:**
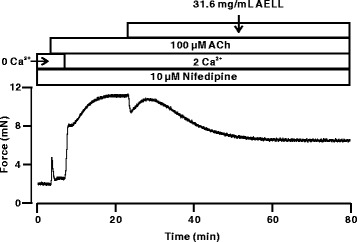
AELL blocks ACh-evoked Ca^2+^ influx. These experiments were performed in the presence of nifedipine (*n* = 4). Under Ca^2+^-free conditions (0 Ca^2+^ and 0.5 mM EGTA), ACh induced a rapid and transient contraction. Following the addition of 2 mM Ca^2+^, a strong sustained contraction occurred, which was partially reversed by AELL

To investigate the role of NSCC in AELL-induced relaxation, Ca^2+^ influx was measured under ACh precontraction (Fig. 5). As shown in Fig. 5, ACh induced a transient contraction in the presence of nifedipine (10 μM) and Ca^2+^-free conditions, which indicates a transient release of intracellular calcium. The addition of 2 mM Ca^2+^ triggered a sustained contraction that was partially blocked (61.8 ± 6.1%) by 31.6 mg/mL AELL. These data indicate that AELL inhibit the nifedipine-resistant Ca^2+^ influx through NSCC.

### AELL Inhibit NSCC Currents

**Fig. 6:**
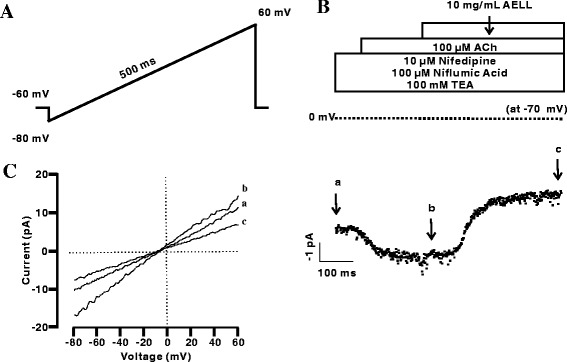
AELL blocks NSCC currents. **a** The protocol used to measure NSCC currents in single ASM cells. **b** *I-t* relationships were recorded at Vm = −70 mV, which indicated that ACh-induced NSCC currents was completely blocked by AELL. **c** The slope currents at time points a, b, and c in (**b**)

To test whether AELL have some effects on NSCC currents, ACh-activated NSCC currents were measured in the presence or absence of AELL. To isolate NSCC currents, nifedipine, NA and TEA were employed to block currents from VDCC, Cl^−^ channels and K^+^ channels, respectively (Fig. 6a). The results show that NSCC currents were completely blocked by AELL (Fig. 6b). Three representative ramp current traces at time points a, b, and c are shown in Fig. 6c. The mean values of the current amplitudes at −70 mV were −15.6 ± 0.5 pA and −7.5 ± 0.5 pA at time points b and c, respectively (*n* = 6, Fig. 6d). Taken together, AELL can inhibit ACh-induced NSCC currents.

## Discussion

As an alternative medicine, the TCM herb has been integrated into orthodox therapies for several chronic diseases associated with the dysfunction of smooth muscles [15, 40]. Furthermore, multiple bioactive constituents from TCM herbs have been identified to have relaxation properties on excessively contractive ASM, which could be complementary therapies for chronic respiratory diseases, especially asthma and COPD [30, 41–46]. In the current study, the relaxant effects of AELL, an alcoholic extraction of *Nelumbo nucifera* leaves, on mouse ASM tone and the underlying electrophysiological mechanisms were explored.

In current asthma therapy, β2-agonists and muscarinic antagonists are two regular bronchodilators, which can result in a quick and lasting relaxant effect. Voltage-dependent calcium channel (VDCC) is involved in β2-agonist-induced tracheal relaxation [47, 48], while VDCC and NSCC both play important roles in muscarinic antagonist-induced ASM relaxation [38, 39]. A high K^+^ concentration causes the contraction of ASM by increasing the intracellular Ca^2+^ concentration, which is induced by plasma membrane depolarization and VDCC activation [35, 36]. ACh, one of the most important neurotransmitters, causes the contraction of ASM by simulating the muscarinic3 receptor, evoking Ca^2+^ influx, which is induced by both VDCC and NSCC opening [38, 39]. Therefore, in the current study, high K^+^ and ACh were employed to stimulate a contractile response in asthma.

It was first investigated whether AELL can relax precontracted ASM by high K^+^ or ACh stimulation (Figs. 1 and 4). It was found that AELL could partially relax high K^+^/ACh-precontracted mouse ASM in a dose-dependent way, and the maximum efficiency of relaxation was approximately 50%. Furthermore, AELL-induced relaxation attained its maximum relaxant effect in 10 min and lasted for hours. All the data confirm that AELL have relaxation characteristics that are similar to those of current bronchodilators.

To further explore the mechanism of AELL-induced relaxation, Ca^2+^ influx and VDCC/NSCC channel currents were studied. Increased Ca^2+^ influx is essential in both high K^+^ and Ach-induced precontraction. Experiments of AELL-induced relaxation were performed with or without Ca^2+^-involved conditions (Figs. 2 and 5). It was found that the relaxant characteristics of AELL were implemented through the inhibition of extracellular Ca^2+^ influx. Although ACh could evoke a transient contraction with intracellular Ca^2+^ release from the sarcoplasmic reticulum as shown in Fig. 5, a sustained contraction occurred only with an extracellular solution including 2 mM Ca^2+^. The results indicate that intracellular calcium influx is indispensable in AELL-induced relaxation.

To demonstrate the roles of VDCC and NSCC in AELL-induced relaxation, VDCC or NSCC currents were measured. It was found that AELL could completely inhibit both VDCC and NSCC currents (Figs. 3 and 6). Considering that AELL did not completely relax high K^+^ or ACh-induced precontraction while VDCC and NSCC currents were both completely blocked, unknown pathways might be involved in AELL-induced relaxation in addition to VDCC and NSCC.

Taken together, our study shows that AELL, an alcoholic extraction of lotus leaves, presents an integrated characteristic of β2-agonists and muscarinic antagonists to relax mouse ASM. Similar to the relaxant mechanism of a regular bronchodilator, Ca^2+^ influx was inhibited, and both VDCC and NSCC were inactivated in AELL-induced relaxation.

The results illuminate the pharmacological characteristics of AELL, which might be a complementary treatment or even an emerging bronchodilator for application in respiratory diseases. However, it should be noted that 17.8 mg/mL or 31.6 mg/mL AELL were employed in the experiment. As a crude extract, the effective component of AELL was unclear. We supposed that the proportion of active ingredient in AELL is not enough to induce a complete relaxation. Meanwhile, some contents of AELL may play a contractive other than relaxant role in ASM. It was also mentioned that the contraction of ASM was hard to recover after rinsing, which indicated that the crude extract might have a high affinity and hard to be totally washed out. However, the type of AELL-induced inhibition (competitive or non-competitive) needs to be further confirmed.

Overall, two issues remain to be resolved. 1. As a crude ethanol extraction, the effective ingredients of AELL are still unknown. Recent studies have revealed that the major constituents of AELL are aporphine alkaloids [49]. Nuciferine, a vital constituent of aporphine alkaloids, had a pharmacologic profile of action associated with arterial relaxation via suppressing extracellular calcium influx [31], which indicates that nuciferine may play an important role in AELL-induced respiratory relaxation and the underlying mechanism needs to be further explored. 2. The partially relaxant characteristics of AELL imply that an unknown pathway might be involved, and the complex intracellular signaling mechanisms require further elucidation.

## Conclusion

AELL directly or indirectly blocked VDCC and NSCC, then reduced calcium influx, and finally relaxed ASM. The exact mechanism of action and the phytochemistry of AELL will be investigated in future studies.

## Additional file

Additional file 1: Figure S1. The influence of AELL on ASM. (A) & (B) With AELL washing out, high K^+^-induced contraction was decreased obviously. (C) & (D) When AELL removed, ACh-induced contraction was decreased obviously, too. (PDF 29 kb)

## Abbreviations

ACh: Acetylcholine chlorides
AELL: Alkaloids extracted from lotus leaves
ASM: Airway smooth muscle
COPD: Chronic obstructive pulmonary disease
LCPSS: Low-Ca^2+^ physiological saline solution
NA: Niflumic acid
NSCC: Non-selective cation channels
PSS: Physiological saline solution
SPF: Specific pathogen free
TCM: Traditional Chinese medicine
TEA: Tetraethylammonium chloride
VDCC: Voltage-dependent Ca^2+^ channels
