# Supplementary figures and images for: Nelumbo nucifera leaves extracts inhibit mouse airway smooth muscle contraction
Source: BMC Complement Altern Med. 2017 Mar 20;17:159. doi: 10.1186/s12906-017-1674-7 (PMC5359798; doi:10.1186/s12906-017-1674-7)

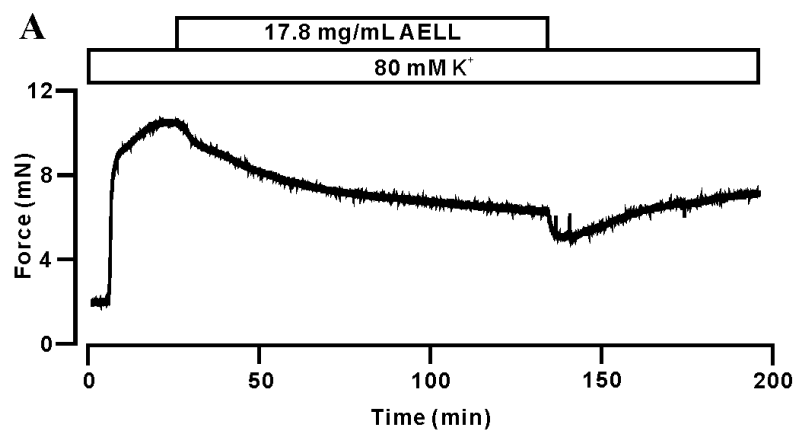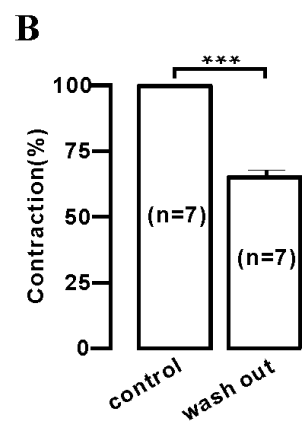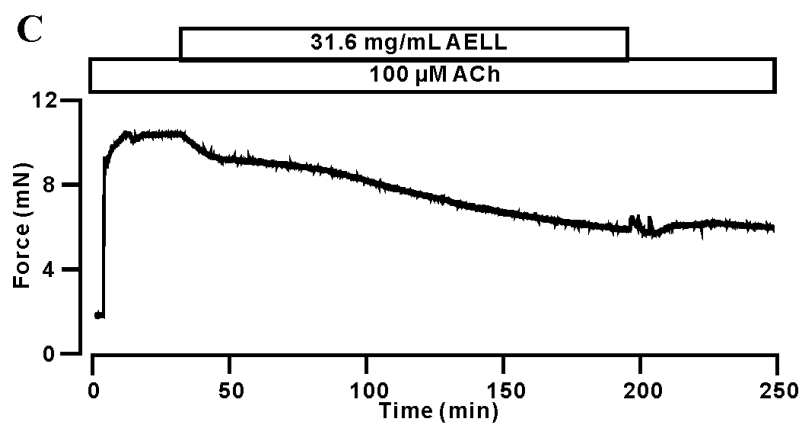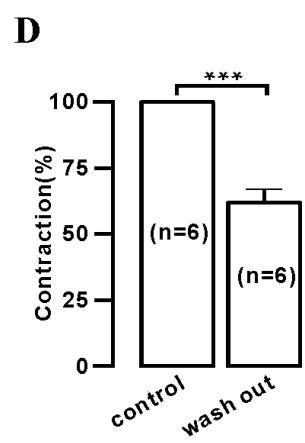

Supplement: Additional file 1: Figure S1. — The influence of AELL on ASM. (A) & (B) With AELL washing out, high K+-induced contraction was decreased obviously. (C) & (D) When AELL removed, ACh-induced contraction was decreased obviously, too. (PDF 29 kb) [file 12906_2017_1674_MOESM1_ESM.pdf]
